# Maternal outcomes of pregnant patients after trauma: a retrospective study of the Trauma Registry of England and Wales

**DOI:** 10.1308/rcsann.2023.0047

**Published:** 2023-08-23

**Authors:** C Demetriou, A Avraam, P Symonds, W Eardley, CB Hing

**Affiliations:** ^1^ East Suffolk and North Essex NHS Foundation Trust, UK; ^2^ School of Medicine, National Kapodistrian University of Athens, Greece; ^3^ Trauma Audit & Research Network, Northern Care Alliance NHS Foundation Trust, UK; ^4^ South Tees Hospitals NHS Foundation Trust, UK; ^5^ St George’s University Hospitals NHS Foundation Trust, UK

**Keywords:** Trauma, Pregnancy, PROMs, EQ-5D, TARN, Registry outcome, Injury, Database

## Abstract

**Introduction:**

Trauma accounts for 20% of deaths in pregnant women. Injury characterisation and outcome in pregnant women following trauma is poorly described. To understand and inform optimum care of this key injury population, a study was conducted using the Trauma Audit Research Network (TARN) database.

**Methods:**

In total, 341 pregnant and 26,774 non-pregnant female patients aged 15 to 46 years were identified for comparison from the TARN database. Mortality, cross-sectional imaging, blood product administration and EQ-5D scores were compared between the two groups. Mechanism of injury, Injury Severity Score (ISS) and mortality rate before and after the creation of regional trauma networks were reported for pregnant patients.

**Results:**

Pregnancy was recorded in 1.3% (341/27,115) of included patients, with the most common cause of injury being road traffic collisions. A reduction in crude maternal mortality was observed over the course of the study period (7.3% to 2.9%). Baseline mean EQ-5D (0.47) and EQ-VAS (54.08) improved to 0.81 (*p* < 0.001) and 85.75 (*p* = 0.001), respectively, at 6 months following injury.

**Conclusion:**

The incidence of trauma in pregnancy is small and mortality in injured pregnant women decreased over the study period. Pregnant patients have significantly improved patient-reported outcome measures 6 months after injury although this is limited in impact because of poor response rates and outcome reporting. Construction and validation of tools aiding in outcome reporting will help considerably in understanding further gains in the care of pregnant women.

## Introduction

Trauma accounts for 140,000 deaths annually in the European Union in people under 45 years of age.^1–3^ A report by the National Confidential Enquiry into Patient Outcome and Death corroborates major trauma as a leading cause of death in patients under 40 years of age, with 75% of those in the report being males under the age of 40 years.^1,4,5^ Similarly, the estimated number of deaths from major trauma each year in England is 5,400.^1,4^ Trauma accounts for 10% of deaths worldwide according to the World Health Organization.^6^ The mortality rate of females in the general population, aged 20–44 years is 117.3 per 100,000 women in the USA.^7^ It is unsurprising, therefore, that injury accounts for up to 20% of maternal deaths relating to non-obstetric causes.^8–11^

A major cause of injury in pregnant women are road traffic collisions (RTCs).^8^ As with other injury populations, falls are similarly causative of trauma in this group.^8,12^ Where trauma in pregnant women differs from other injury populations is intimate partner violence, a key and potentially poorly understood domain of trauma care.^8,13–15^

RTCs (55.5%), falls (31.8%) and assault (11%) were noted as causative factors by Battaloglu *et al* in their study of 15,140 patients in the United Kingdom (UK) over a 6-year period.^16^ An overall mortality rate of 5.1% was reported in this study,^16^ which preceded the routine collection of outcome measures and so offers context for numbers but lacks outcome reporting.

Mortality in injured pregnant women is variably described owing to the heterogeneity of reporting methods and injury mechanisms. For example, in this group, mortality resulting from RTCs has been reported as being 0.04%^17^and 0.001%.^18^

The Trauma Audit Research Network (TARN) has used the EuroQol questionnaire (EQ-5D–5L) for patients admitted to a major trauma centre (MTC) at baseline and 6 months following injury since 2014.^19^ The aim of the current study was to identify the incidence of pregnancy in trauma reported in the TARN registry, assess the mortality of pregnant women reported in the TARN registry and investigate outcome assessment and reporting using patient-reported outcome measures (PROMs) for the first time in the UK.

## Methods

TARN collects data on patients sustaining major trauma in England and Wales, Ireland and some hospitals from Continental Europe. TARN includes patients who are admitted to hospital for three or more days, require critical care resources, are transferred for further care or die from their injuries. Isolated injuries, including fractures of the pubic ramus and proximal femur in patients aged over 65 years or isolated closed limb fractures, are specifically excluded. A search was performed from inception of the TARN database (January 1989) to 31 December 2021 to identify pregnant patients aged under 50 years as well as an age-matched non-pregnant cohort.

The following parameters were recorded: age, Injury Severity Score (ISS), Abbreviated Injury Scale (AIS), cross-sectional imaging, use of blood products and EQ-5D–5L scores. Patient injuries were subdivided into six anatomic regions, and each was assigned a score according to the AIS. For multiple injuries in the same anatomic region, the highest score was considered. ISS was calculated as the sum of squares of the three highest AIS scores, with each score corresponding to a different anatomic region. ISS was recorded for each patient, with a range of 1–75.^20,21^

### Data analysis

Approval was given to TARN by the UK Health Research Authority Patient Information Advisory Group to perform research on anonymised data in the TARN registry.

The mechanism of injury and ISS were reported for pregnant patients. Mortality and survival rates were compared between pregnant and non-pregnant patients. The use of blood products for resuscitation and whether the patient had undergone a trauma computed tomography scan (CT) was compared between the two groups. Comparison of the mortality rates for pregnant patients treated before establishment of the trauma network and after was carried out.

EQ-5D–5L questionnaire responses were converted to the EQ-5D–5L score with a minimum score of −0.285 and maximum score of 1. For this purpose, we used the UK data valuation set and the scoring conversion table by Devlin *et al*.^22^ The EQ-5D visual analogue scale score from 0 to 100 was also collected for patients who responded to the questionnaire. The baseline questionnaire was filled in by patients during their acute admission. The 6-month questionnaire was sent to the patients by post. Comparison of the baseline and 6-month mean scores was performed for pregnant and non-pregnant patients who responded to the questionnaire. Pregnant and non-pregnant patients who responded to the EQ-5D questionnaire were matched on the AIS score of the six anatomical regions. Comparison of the mean EQ-5D scores of matched pregnant patients was performed for each category of ISS (1–8, 9–15, >15).

Descriptive statistics were calculated for the variables presented in the Results section. Percentages along with the number of cases are reported for the categories in Tables 1–5*.* The mean, standard deviation (sd) and range are reported for continuous variables (age, EQ-5D score and EQ-VAS score). The chi-squared test was used to compare categorical variables (mortality, CT scan and blood products), and the *t*-test was used for continuous variables (EQ-5D and EQ-VAS scores). Odds ratios (OR) of death were calculated for the AIS in pregnant patients who survived and those who died. The level of statistical significance was set at *p* < 0.05. Statistical analysis was performed in SPSS version 22.

**Table 1:** Mechanism of injury, Injury Severity Score (ISS) for pregnant patients

Mechanism of injury	Pregnant (*n* = 341)
Vehicle collision	201 (58.9%)
Fall >2m	50 (14.7%)
Fall <2m	40 (11.7%)
Stabbing	19 (5.6%)
Shooting	3 (0.9%)
Blow without weapon	20 (5.9%)
Other	8 (2.3%)
Blunt	315 (92.4%)
Penetrating	26 (7.6%)
Injury Severity Score	
1–8	65 (19.1%)
9–15	93 (27.3%)
>15	183 (53.7%)

**Table 5:** Mean EQ-5D–5L score and EQ-VAS score for pregnant and non-pregnant patients both at baseline and six months after admission

	Pregnant	Non-pregnant	*p*-value
EQ-5D score at baseline	0.47 (–0.23 to 0.83), sd (0.25), *n* = 17	0.44 (–0.29 to 1.00), sd (0.30), *n* = 1573	
EQ-5D score for matched patients with ISS 1–8 at baseline	0.62 (0.49 to 0.83), sd (0.19), *n* = 3	0.34 (–0.29 to 0.74), sd (0.32), *n* = 32	0.16
EQ-5D score for matched patients with ISS 9–15 at baseline	0.33 (–0.22 to 0.62), sd (0.18), *n* = 6	0.45 (–0.01 to 0.87), sd (0.21), *n* = 65	0.18
EQ-5D score for matched patients with ISS >15 at baseline	0.52 (0.30 to 0.78), sd (0.18), *n* = 8	0.53 (–0.03 to 0.88), sd (0.25), *n* = 26	0.91
EQ-5D score six months after admission	0.81 (0.67 to 1.00), sd (0.13), *n* = 5	0.66 (–0.29 to 1.00), sd (0.28), *n* = 513	
EQ-5D score for matched patients with ISS 1–8 six months after admission	1.00, *n* = 1	0.92, *n* = 1	
EQ-5D score for matched patients with ISS 9–15 six months after admission	0.74 (0.67 to 0.81), sd (0.10), *n* = 2	0.61 (–0.04 to 1.00), sd (0.27), *n* = 61	0.51
EQ-5D score for matched patients with ISS >15 six months after admission	0.77 (0.71 to 0.83), sd (0.09), *n* = 2	0.82 (0.68 to 1.00), SD (0.17), *n* = 3	0.77
EQ-VAS score at baseline	54.08 (8 to 100), sd (25.20)	50.69 (0 to 100), sd (23.00)	
EQ-VAS score six months after admission	85.75 (80 to 100), sd (9.60)	66.26 (0 to 100), sd (21.40)	

ISS = Injury Severity Score

## Results

**Figure 1:**
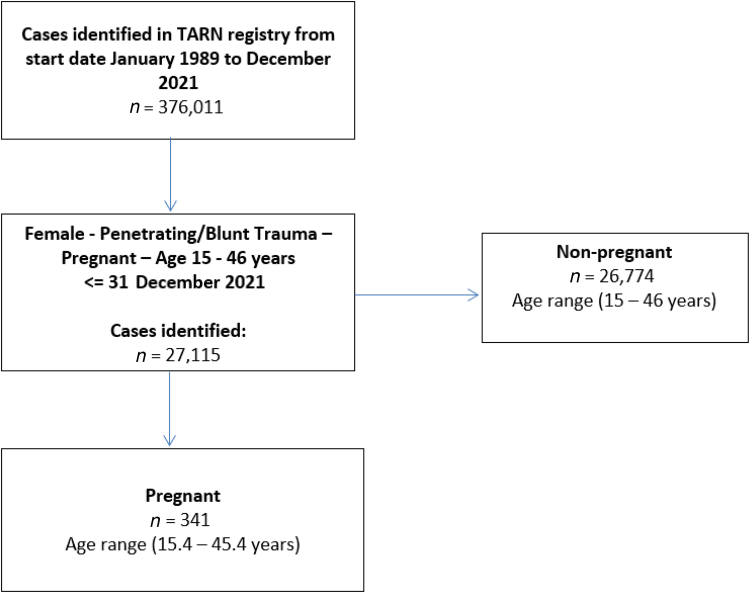
Search strategy used to identify the patients in the Trauma Audit Research Network registry

A search of the TARN registry database identified 341 pregnant patients, mean age 28.7 years (15.4 to 45.4, sd = 6.7) from 27,115 female patients aged 15–46 years (Figure 1). The mean age of non-pregnant patients (*n* = 26,774) was 30.3 years (sd = 9.2). The incidence of pregnancy in female trauma patients in this age group from the registry was 1.3%. From January 1989 to December 2021, data for 376,011 female patients were reported in the registry, thus the overall incidence of pregnancy in trauma was 0.09%.

Table 1 summarises the mechanisms of injury and the ISS for the pregnant patients, with RTCs and falls being the commonest and 53.7% of the pregnant patients sustaining major trauma (ISS > 15). The mortality rate for pregnant patients is 4.1% (14 of 341) compared with 5.5% (1,462 of 26,774) for non-pregnant patients. There is no evidence of a difference in mortality between pregnant and non-pregnant patients (χ^2^ = 1.20, risk ratio = 1.01; 95% confidence interval [CI] 0.99–1.03, *p* = 0.27). The baseline distribution of the ISS for the non-pregnant patients is shown in Table 2. We identified 26,431 non-pregnant patients who matched the pregnant patients’ ISS. The mortality rate of the matched non-pregnant patients is 5.5%. There is no significant difference in mortality between pregnant and matched non-pregnant patients (χ^2^ = 1.28, *p* = 0.26). The mechanisms of injury for pregnant patients who died are shown in Table 3. The ISS for pregnant patients who did not survive trauma was 9–15 for 1 patient and >15 for the remaining 13 patients. Pregnant patients who died were much more likely to sustain severe intracranial, thoracic, abdominal and pelvic injuries, with only thoracic trauma having the highest and statistically significant OR of death of 5.37 as shown in Table 3. The mean age of the pregnant patients who died was 28.1 years with the youngest aged 17.3 years and the oldest aged 39.9 years (sd = 6.2). The mean age of the living pregnant patients was 28.7 years (sd = 6.7) and there was no significant difference from the dead pregnant patients (*p* = 0.37).

**Table 2:** Mean age and Injury Severity Score (ISS) for non-pregnant patients who died and number of pregnant patients who died matched to non-pregnant patients who died according to exact ISS

ISS	Non-pregnant patients (*n* = 26,774; mean age = 30.3 years, sd = 9.2, range 15–46)	
1–8	4,855 (18.1%)	
9–15	8,935 (33.4%)	
>15	12,984 (48.5%)	
ISS	Pregnant patients (*n* = 341; mean age = 28.7 years, sd = 6.7, range 15.4–45.4)	Matched non-pregnant patients (*n* = 26,431; mean age = 30.3 years, sd 9.2, range 15–46)
9–15	1 (0.3%)	56 (0.2%)
>15	13 (3.8%)	1,400 (5.3%)

**Table 3:** Mechanisms of injury of pregnant patients who died because of trauma and odds ratio of death and distribution of alive and dead pregnant patients, with Abbreviated Injury Scale (AIS) >3 (severe injury) in each anatomical region of the Injury Severity Score (ISS)

Injury mechanism	Number		
Road traffic accident	9 (64.3%)		
Shooting	2 (14.3%)		
Stabbing	1 (7.1%)		
Fall >2m	1 (7.1%)		
Other	1 (7.1%)		
Anatomical area	Alive pregnant patients with ISS > 15 (*n* = 170)	Dead pregnant patients with ISS > 15 (*n* = 13)	Odds ratio (95% confidence intervals)
Head AIS >3	57 (33.5%)	6 (46.2%)	1.70 (0.45–6.20)
Chest AIS >3	39 (22.9%)	8 (61.5%)	5.37 (1.44–21.88)
Abdomen AIS >3	22 (12.9%)	3 (23.1%)	2.02 (0.33–8.45)
Pelvis AIS >3	28 (16.5%)	4 (30.8%)	2.25 (0.45–8.75)

AIS = Abbreviated Injury Scale

The mortality rate for pregnant patients was 7.3% (7/96) before creation of the regional trauma networks (RTNs) and 2.9% (7/245) after (χ^2^ = 3.45, *p* = 0.063).

Pregnant patients (242/341, 71%) were as likely to undergo a trauma CT scan on admission as non-pregnant patients (19,603/26,774, 73.2%; χ^2^ = 0.87, *p* = 0.35). Blood products were administered to 54 (15.8%) pregnant patients for resuscitation compared with 2,193 (8.2%) non-pregnant patients (χ^2^ = 25.89, *p* < 0.001).

The collection of PROMs (EQ-5D–5L) by the TARN registry was introduced in 2014. Table 4 summarises the response rate for eligible pregnant and non-pregnant patients along with the respective ISS and severity of injuries for each group both at baseline and 6 months after admission. Table 5 summarises the mean EQ-5D and EQ-VAS scores for pregnant and non-pregnant patients at baseline and 6 months after admission. The mean EQ-5D score was significantly improved 6 months after admission compared with that at baseline, both for pregnant (*p* < 0.001) and non-pregnant (*p* < 0.001) patients. In addition, the mean EQ-VAS score was significantly improved 6 months after admission both for pregnant (*p* = 0.001) and non-pregnant (*p* < 0.001) patients compared with the mean EQ-VAS score at baseline. When comparing the mean EQ-5D scores for matched pregnant and non-pregnant patients based on the AIS per anatomical area, there was no statistically significant difference between the categories ISS 1–8, 9–15 and >15 at baseline (*p* = 0.16, 0.18, 0.91) and 6 months after admission (*p* = 0.51, 0.77) (Table 5). There was only one EQ-5D score reported at 6 months for pregnant patients with ISS 1–8, which was matched to only one non-pregnant patient (1 vs 0.92 respectively).

**Table 4:** Response rates to EQ-5D–5L questionnaire for pregnant and non-pregnant patients at baseline and six months after admission along with respective Injury Severity Score (ISS) distribution for each group

	Pregnant (*n* = 217)	Non-pregnant (*n* = 14,827)
Response rate at baseline (on admission)	17 (7.8%)	1,573 (10.6%)
ISS 1–8	3 (17.6%)	175 (11.1%)
ISS 9–15	6 (35.3%)	464 (29.5%)
ISS >15	8 (47.1%)	934 (59.4%)
Response rate six months after admission	5 (2.3%)	513 (3.5%)
ISS 1–8	1 (20%)	53 (10.3%)
ISS 9–15	2 (40%)	151 (29.4%)
ISS >15	2 (40%)	309 (60.2%)

No data were routinely collected by the TARN registry of the fetal outcome for the pregnant patients who sustained trauma and were reported to the registry. Therefore, we cannot comment on the management and survival of the fetus from these data.

## Discussion

The occurrence of pregnancy in major trauma patients is low. The most common mechanisms of injury in pregnant patients, in order of decreasing frequency are: vehicle collision, falls, stabbing, shooting, blow without weapon and other. There is a much higher prevalence of blunt compared with penetrating trauma. This agreed with Battaloglu *et al* who reported the same mechanisms of injury and a similar incidence rate of pregnancy in trauma from the TARN registry between 2009 and 2014.^16^ El Kady *et al* reported a rate of 0.2% for trauma in pregnancy in the state of California between 1991 and 1999.^17^ The mechanisms of injury mentioned in El Kady *et al* in order of decreasing frequency were: falls, RTCs, assault, shooting, burns and suicide attempts.^17^

The mortality rate of 4.1% is lower than the 5.1% reported by Battaloglu *et al.*^16^ Mitra *et al* reported that trauma in pregnancy was responsible for 9% (2,605/29,088 maternal deaths) of deaths in pregnancy between 1979 and 2017 in the USA.^10^ Moran *et al* reported that since the establishment of RTNs in April 2012, the outcomes for trauma patients had improved, including survival of patients with major trauma.^1^ Despite being statistically insignificant, there is still a reduction in the mortality rate in pregnant women from 7.3% before the creation of RTNs to 2.9% after. The small number of pregnant patients recorded in the TARN registry may have led to this result being insignificant. One explanation for this improvement is that the collective expertise of different specialties in dealing with complex major trauma patients available in MTCs has improved the outcomes for pregnant patients as well as other major trauma patients. Advances in the care of major trauma patients have also significantly contributed to the improved mortality rate.

Pregnant patients who die are more likely to have severe thoracic, pelvic, abdominal and intracranial injuries. This is in agreement with El Kady *et al*, who reported higher incidences of thoracic, abdominal and pelvic injuries followed by intracranial injuries as leading causes of death in pregnant patients.^17^

Pregnant patients had a similar rate of trauma CT scans performed and increased administration of blood products for resuscitation compared with non-pregnant patients. It is difficult to comment on these trends; for example, whether this was justified from the clinical presentation and/or physiological parameters of the patients. However, exposure to significant radiation and the use of blood products should be done with caution to avoid unnecessary harm to the fetus.

Jain *et al* have advised that if an abdominal CT scan is required for the evaluation of a pregnant patient, this should not be deferred owing to concerns of radiation exposure to the fetus because the lifetime cancer risk from prenatal exposure is thought to be quite low.^11^ They also recommended if an urgent blood transfusion is required O negative blood should be given to pregnant patients until a crossmatch is available to avoid Rhesus D alloimmunisation.^11^

In our study, pregnant patients report significant improvement 6 months after admission in their PROMs (Table 5). There was no significant difference in the mean EQ-5D scores between pregnant and non-pregnant patients matched for injury severity and anatomical region of injury.

There are multiple minimum clinically important differences (MCIDs) for the EQ-5D score reported in the literature. Walters *et al* calculated a mean MCID for EQ-5D of 0.074 and a median MCID of 0.081.^23^ The improvement of 0.34 seen in the mean EQ-5D score for pregnant patients, 6 months from admission, is well above the MCID and hence is clinically important. MCID for the EQ-VAS score was calculated by Yapp *et al* at 6.41.^24^ The improvement of 31.67 in the mean EQ-VAS score for pregnant patients, 6 months from admission, is also above the reported MCID; therefore, patients will notice a difference in their activities of daily living and symptoms.

To our knowledge, this is the first time that EQ-5D and EQ-VAS scores have been reported for pregnant patients who sustained trauma, so we do not have a comparator.

### Study limitations

This is a retrospective study that relied on the reporting of data from centres participating in the TARN registry. Particularly useful data relating to trauma in pregnancy (physiological data of the women at the time of presentation, risk pregnancy status, semester of pregnancy, fetal survival, administration of anti-D immunoglobulins following blood product transfusion or placental disruption and involvement of obstetrics) are not routinely collected by TARN and are not readily available within the database. Hence, it is difficult to comment on the effect of those parameters on the management of pregnant patients. Minor injuries in pregnancy are not reported to the TARN registry, thus our study is under-reporting the incidence of trauma in pregnancy. More data need to be collected across multiple trauma centres and prospective studies performed to comment on those parameters. Another limitation is that the TARN registry does not routinely record data about the management and outcomes of the fetuses involved. Thus, we are not able to comment on any fetal outcomes in this study.

The response rate to the EQ-5D questionnaires is low, making it difficult to reliably calculate a good estimate of the mean scores and use regression analysis to identify factors influencing the outcomes of given care. Physiological data and observations at the time of presentation, as well as the type of definitive treatment provided, are not available, making it hard to compare a matched cohort of pregnant with non-pregnant patients. The response rate could be improved if regular reminders were sent to the patients and/or the questionnaires were delivered electronically. Repeat questionnaires at 1 and 2 years after submission would establish whether patients with major trauma (ISS > 15) continue to improve.

## Conclusions

The occurrence of pregnancy in trauma patients is low. The mortality rate of pregnant patients has decreased over the study period. As reported in PROMs, pregnant patients with trauma have significantly improved outcomes 6 months after admission compared with baseline. Further prospective studies are required to assess management of these patients and achieve a higher response rate to PROMs for analysis of outcome-determining factors.
